# Use of Changestat for Growth Rate Studies of Gut Microbiota

**DOI:** 10.3389/fbioe.2020.00024

**Published:** 2020-02-07

**Authors:** Kaarel Adamberg, Grete Raba, Signe Adamberg

**Affiliations:** ^1^Department of Chemistry and Biotechnology, Tallinn University of Technology, Tallinn, Estonia; ^2^Center of Food and Fermentation Technologies, Tallinn, Estonia

**Keywords:** continuous cultivation, changestat, fecal microbiota, apple pectin, birch xylan

## Abstract

Human colon microbiota, composed of hundreds of different species, is closely associated with several health conditions. Controlled *in vitro* cultivation and up-to-date analytical methods make possible the systematic evaluation of the underlying mechanisms of complex interactions between the members of microbial consortia. Information on reproducing fecal microbial consortia can be used for various clinical and biotechnological applications. In this study, chemostat and changestat cultures were used to elucidate the effects of the physiologically relevant range of dilution rates on the growth and metabolism of adult fecal microbiota. The dilution rate was kept either at *D* = 0.05 or *D* = 0.2 1/h in chemostat cultures, while gradually changing from 0.05 to 0.2 1/h in the A-stat and from 0.2 to 0.05 1/h in the De-stat. Apple pectin as a substrate was used in the chemostat experiments and apple pectin or birch xylan in the changestat experiments, in the presence of porcine mucin in all cases. The analyses were comprised of HPLC for organic acids, UPLC for amino acids, GC for gas composition, 16S-rDNA sequencing for microbial composition, and growth parameter calculations. It was shown that the abundance of most bacterial taxa was determined by the dilution rate on both substrates. *Bacteroides ovatus*, *Bacteroides vulgatus*, and *Faecalibacterium* were prevalent within the whole range of dilution rates. *Akkermansia muciniphila* and Ruminococcaceae UCG-013 were significantly enriched at *D* = 0.05 1/h, while *Bacteroides caccae*, Lachnospiraceae unclassified and *Escherichia coli* clearly preferred *D* = 0.2 1/h. In the chemostat cultures, the production of organic acids and gases from pectin was related to the dilution rate. The ratio of acetate, propionate and butyrate was 5:2:1 (*D* = 0.05 1/h) and 14:2:1 (*D* = 0.2 1/h). It was shown that the growth rate-related characteristics of the fecal microbiota were concise in both directions between *D* = 0.05 and 0.2 1/h. Reproducible adaptation of the fecal microbiota was shown in the continuous culture with a changing dilution rate: changestat. Consortia cultivation is a promising approach for research purposes and several biotechnological applications, including the production of multi-strain probiotics and fecal transplantation mixtures.

## Introduction

The cultivation of fecal consortia is essential for understanding the mechanisms behind the coexistence of gut microbial species under changing environmental conditions. This information is required in biotechnological and clinical applications, although better defined methods are required for safer procedures. As the desired consortia can be composed of tens or even hundreds of strains, e.g., next generation probiotics, the cultivation of a balanced consortium saves time and labor compared to the production of single cultures. Moreover, the biomass yields of pure cultures can remain below those of natural bacterial communities, because of a deficiency of several growth factors produced by other consortia members. In this field, the culturomics approach has been developed, where a set of different media are used to cultivate each isolate in a multi-parallel approach (Lagier et al., 2012). This approach has expanded the range of microorganisms that can be cultivated in the lab. The reintroduction of fecal microbiota is a promising method to cure certain gastrointestinal conditions (Petrof et al., 2013). Previously, the most diverse stool substitute containing 33 single fecal isolates was developed by Petrof et al. (2013) to cure antibiotic-resistant *Clostridium difficile-*induced colitis. Also, batch cultures have been used for the production of fecal biomass to treat diarrhea caused by *C. difficile* infection (Jorup-Rönström et al., 2012). Batch cultures are most commonly used for high-throughput small-scale parallel experiments. However, such conditions as substrate concentrations and the accumulation of metabolites are continuously changing. Furthermore, the overgrowth of fast-growing bacteria in mixed cultures is common for batch cultures; for instance, an over 10% increase in *Escherichia coli* from the total population has been reported (Brahma et al., 2017; Adamberg et al., 2018). Thus, it is difficult to analyze the actual selectivity of a tested substrate. Consequently, batch technologies are well suited for high throughput screening or for the industrial production of biomass, but have limited value for studies of specific growth mechanisms and the metabolism of complex microbial consortia.

In a microbial consortium, the steady state composition is defined by complex microbial interactions. These interactions can be supportive (mutual or commensal) or inhibitory (ammensal or competitive), and are driven by residual concentrations of substrates, bacterial metabolites and cross-feeding between different bacteria (Gottschal, 1990). By cultivating 37 mouse gut bacteria in continuous mode, Freter et al. (1983) demonstrated that the population dynamics of indigenous intestinal bacteria are controlled by one or a few substrates. Chung et al. (2019) studied the degradation of five different fibers or fiber mixtures by fecal microbiota in a chemostat and showed that mixed fiber substrates led to the growth of more diverse microbiota than inulin alone. In the cultures of gut microbes, cross-feeding has been supposed to be one of the most important factors for gut microbiota richness. To explain the interactions between different microorganisms in a consortium, in addition to cell modeling, simple culture systems, such as defined mixed cultures and single substrates, should be studied first. This makes it possible to elucidate the primary degraders and substrates (hetero- or autotrophies), auxotrophies and compounds derived from cross-feeding. The dynamic data for predicting bacterial behavior in communities can be best obtained from continuous cultures at low substrate concentrations (Russell and Baldwin, 1979; Tannock, 2017). For example, ecological studies have shown that growth under multiple substrate limitations at very low dilution rates supports species having high growth efficiency (higher yield) but low maximal specific growth rate (Gottschal, 1990). Auxotrophy to a specific compound can be used to promote a species in a mixed culture by supplementing the culture medium with this substrate. Two species containing batch experiments revealed that acetate or lactate produced by *Bacteroides* or *Bifidobacterium* stimulated the growth of butyric acid producing bacteria, while formate and hydrogen enhanced methanogens (Rowland et al., 2018). The dynamics and stability of freshly collected fecal cultures have been studied by several groups, although the use of fresh samples does not allow for direct comparison of the results from different studies (Miller and Wolin, 1981; Macfarlane et al., 1998; Sghir et al., 1998; McDonald et al., 2013; Yen et al., 2015; Chung et al., 2016; von Martels et al., 2017).

The determination of community composition is essential in microbiota research. Next-generation sequencing methods, such as 16S rDNA analysis and whole genome sequencing (WGS), are high throughput approaches that make it possible to identify all of the taxa in whole consortia, but only semi-quantitatively as proportions of the bacteria in a consortium. To obtain quantitative data, bacterial counting through flow cytometry, dry weight analysis or plate counting should be carried out in parallel. Moreover, to determine the taxa of low abundance, the coverage of sequences has to be proportionally higher. Also, species level analysis might require more detailed sequence analysis (WGS) than is available by 16S rDNA sequencing. For the quantitative analysis of bacteria in fecal consortia, species are usually assessed by fluorescent *in situ* hybridization with 16S rRNA probes (Langendijk et al., 1995), although as each species requires a specific probe, this approach is limited by the number of species analyzed or is expensive in an array setup, such as HITChip (Rajilić-Stojanović et al., 2009).

With all cultivation models it should be kept in mind that the specific growth rate of bacteria is not linearly related to the colonic transit rate, since the density of bacteria gradually increases, while the moving rate decreases along the colon. An alternative continuous cultivation technology, changestat, in which all cultivation parameters are computer-controlled, has been developed in our lab. In changestat, the effect of a selected parameter is studied by the gradual change in this parameter within a certain range, while keeping all other conditions constant (Paalme et al., 1995; Kasemets et al., 2003; Adamberg et al., 2015). Our recent study highlighted the importance of dilution rate in determining the composition and diversity of fecal microbiota (Adamberg and Adamberg, 2018). It was also shown that by using de-celerostat (De-stat), the fast- and slow-growing consortia were differentiated from the fecal microbiota during the same experiment (Adamberg and Adamberg, 2018). To analyze whether these results were too biased depending on the starting point, we carried out experiments in both directions: gradually moving dilution rates from slow to fast (accelerostat: A-stat) and from fast to slow (De-stat), between 0.05 and 0.2 1/h.

The main aims of the current study were: (1) to elucidate the reproducibility of the chemostat cultures by using the same adult fecal pool, (2) to study the effects of dilution rate on the dynamics and metabolism of fecal microbiota by using A-stat and De-stat cultures, and (3) to elucidate the metabolism of two common dietary fibers, pectin and xylan, by fecal microbiota. Food is an important factor in modulating colonic microbiota and through bacterial metabolism promoting health-supporting or disease-activating mechanisms. Pectins are a part of the daily diet, consumed in the form of fruits and vegetables and used as food additives. Xylans are abundant in nature as major constituents of hemicellulose in plant cells.

## Materials and Methods

### Fecal Inoculum

Fecal samples were collected from seven healthy adult volunteers (19–37 years old, Caucasian, both male and female) and homogenized in four volumes of 5% DMSO-containing buffer, as described in Adamberg et al. (2015). The exclusion criteria included the use of supplements of prebiotics and probiotics, laxatives and antibiotics for 4 weeks prior to donation. Equal volumes of the seven fecal slurries were pooled together and aliquots were kept at −80°C for repetitive cultivation experiments. Similar sample preparation (standardization by pooling) has also been used and approved by others for *in vitro* testing in the TIM-2 proximal colon model (Aguirre et al., 2015; Bussolo de Souza et al., 2019).

### Defined Base Medium

The defined growth medium was prepared in a 0.05 M potassium phosphate buffer made from 1 M stock solutions (ml/L): K_2_HPO_4_ (28.9) and KH_2_PO_4_ (21.1); mineral salts (mg/L): MgSO_4_^∗^7H_2_O (36), FeSO_4_^∗^7H_2_O (0.1), CaCl_2_ (9), MnSO_4_^∗^H_2_O (3), ZnSO_4_^∗^7H_2_O (1), CoSO_4_^∗^7H_2_O (1), CuSO_4_^∗^5H_2_O (1), (NH_4_)_6_Mo_7_O_24_^∗^4H_2_O (1), NaCl (527); hemin (5 mg/L); vitamin K1 (0.5 mg/L); L-amino acids (g/L): Ala (0.044), Arg (0.023), Asn (0.038), Asp (0.038), Glu (0.036), Gln (0.018), Gly (0.032), His (0.027), Ile (0.060), Leu (0.120), Lys-HCl (0.080), Met (0.023), Phe (0.050), Pro (0.041), Ser (0.095), Thr (0.041), Trp (0.009), Val (0.060), Tyr (0.015); vitamins (mg/L): biotin (0.25), Ca-pantothenate (0.25), folic acid (0.25), nicotinamide (0.25), pyridoxine-HCl (0.50), riboflavin (0.25), thiamine-HCl (0.25) and other components (g/L): bile salts (0.5), NaHCO_3_ (2.0), Tween-80 (0.5), Na-thioglycolate (0.5), and Cys-HCl (0.5, freshly made). The carbohydrate substrates were sterilized separately and mixed with the medium before cultivation. Two substrate combinations, either birch xylan (Sigma-Aldrich, United States) or apple pectin (Sigma-Aldrich, United States) with porcine mucin (Type II, Sigma Aldrich, United States), were added to the base medium in equal amounts (2.5 g/L each). The pH of the growth medium was 7.2 ± 0.1.

### Fermentation System

The Biobundle cultivation system consisted of the ADI 1030 bio-controller and cultivation control program “BioXpert” (Applikon, Netherlands). The fermenter was equipped with sensors for pH, pO_2_, and temperature. Variable speed pumps for feeding and outflow were controlled by a De-stat or A-stat algorithm: D = D_0_ – d^∗^t or D = D_0_ + a^∗^t, respectively, where D is the dilution rate (1/h), D_0_ is the initial dilution rate, d and a are the deceleration and acceleration rate (1/h^2^), respectively, and t is the time (h). In accelerostat (A-stat), the dilution rate was gradually increased from 0.05 to 0.2 1/h and in decelerostat (De-stat) the dilution rate was gradually decreased from 0.2 to 0.05 1/h in accordance with the typical transit rate of the human colon (Adamberg and Adamberg, 2018). pH was controlled by a 1M NaOH addition according to the pH set-point. The culture volume was kept constant (300 mL) by monitoring the weight of the fermenter with the PC-linked balance and outflow pump. The pH of the culture was kept at 7.0 and the temperature was kept constant at 36.6°C. The medium in the feeding bottle and the fermenter was flushed with sterile-filtered nitrogen gas (99.9%, AGA) overnight before inoculation and throughout the cultivation to maintain anaerobiosis. Nitrogen flushing was on during the whole experiment. Two mL of the pooled fecal culture was inoculated to start the experiment.

**FIGURE 1:**
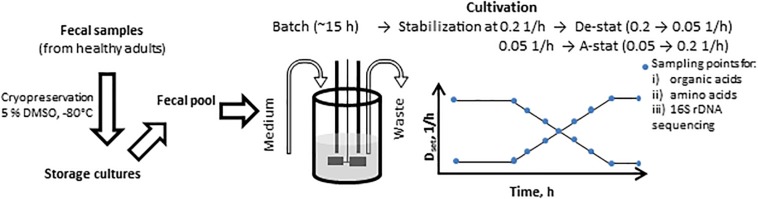
Scheme of the A-stat (accelerostat) and De-stat (decelerostat) experimental set-up. The pooled fecal inoculum was added into 300 ml growth medium at time 0 h followed by batch phase (ca 15 h) until mid- exponential growth phase. Then continuous mode was started and after the stabilization of the fecal culture at *D* = 0.2 1/h (De-stat) or *D* = 0.05 1/h (A-stat, 6-7 residential times in total), the dilution rate was gradually decreased down to 0.05 1/h or increased up to 0.2 1/h (deceleration or acceleration rate 0.05 1/h per day), respectively, followed by re-stabilization for approximately 2 residential times. The same procedure was applied for two substrate combinations (birch xylan + mucin and, apple pectin + mucin). D_set_ indicates the controlled change of the dynamics of the pre-set dilution rate.

The cultivation algorithm was started 15–17 h after inoculation in the midst of the exponential growth of bacteria. The dilution rate was stabilized at either 0.05 or 0.2 1/h, at pH 7.0, and run for stabilization at these conditions by 6–7 residence times. After achieving a stable titration rate and gas production, the dilution rate was decreased to 0.05 1/h or increased to 0.2 1/h at a rate of 0.05 units per day (the experimental timeline is presented in Figure 1). The dilution rate interval was chosen to cover the realistic growth rates of luminal bacteria in the colon. The range of the specific growth rate of the bacteria was calculated based on the colonic transit time of digesta in people consuming Western diets, which varies from 40 to 140 h (median 60–70 h) (Burkitt et al., 1972; Cummings et al., 1976; Fallingborg et al., 1989), and the estimated amount of bacteria, which increases from 10^8^ in the proximal colon to 10^11^ cfu/g in feces (Sender et al., 2016). Considering both the period the bacteria have for degradation of dietary fibers and the coinciding increase in the bacterial biomass in the colon, the specific growth rate of the bacteria decreased from 0.3 to 0.02 1/h. Thus, the range of the dilution rates tested in the cultivation experiments, 0.05–0.2 1/h, was chosen.

### Analytical Methods

Samples from the outflow were collected on ice, centrifuged (14,000 g, 5 min, 4°C) and stored separately as pellets and supernatants at −20°C until HPLC analyses (sugars and organic acids), UPLC analyses (amino acids), and microbial 16S rDNA sequencing were carried out.

For chromatographic analyses, culture supernatants were filtered using AmiconR Ultra-10K Centrifugal Filter Devices, cut-off 3 kDa according to the manufacturer’s instructions (Millipore, United States). The concentrations of organic acids (succinate, lactate, formate, acetate, propionate, isobutyrate, butyrate, isovalerate, and valerate), ethanol and free sugars (mono-, di-, and trisaccharides) were determined by high-performance liquid chromatography (HPLC, Alliance 2795 system, Waters, Milford, MA, United States), using BioRad HPX-87H column (Hercules, CA, United States) with isocratic elution of 0.005 M H_2_SO_4_ at a flow rate of 0.5 mL/min and at 35°C. Refractive index (RI) (model 2414; Waters, United States) and UV (210 nm; model 2487; Waters, United States) detectors were used for quantification of the substances. The detection limit for the HPLC method was 0.1 mM. Concentrations of amino acids and amines were determined with an amino acid analyzer (UPLC; Waters, Milford, United States) according to the manufacturer’s instructions. The detection limit of the method was 0.01 mM. All standard substrates were of analytical grade. Empower software (Waters, United States) was used for the processing of HPLC and UPLC data.

The composition of the gas outflow (H_2_, CO_2_, H_2_S, CH_4_, and N_2_) was analyzed using an Agilent 490 Micro GC Biogas Analyzer (Agilent 269 Technologies Ltd., United States) connected to a thermal conductivity detector. The volume of the gas flow was regularly recorded.

The Redox potential of the growth medium and culture supernatant was measured by a pH/Redox meter using an InLab^®^ Redox electrode (Mettler Toledo).

The biomass dry weight was measured gravimetrically by centrifuging the biomass from a 10 mL culture, washing twice with distilled water and drying in an oven at 105°C for 24 h.

### DNA Extraction and Amplification

DNA was extracted from the pellets using a PureLink Microbiome DNA extraction kit (Thermo Fisher Scientific, United Kingdom) according to the manufacturer’s instructions. Universal primers:

S-D-Bact-0341-b-S-17 Forward (5′TCGTCGGCAGCGTCAG ATGTGTATAAGAGACAGCCTACGGGNGGCWGCAG) and S-D-Bact-0785-a-A-21 Reverse (5′GTCTCGTGGGCTCGGA GATGTGTATAAGAGACAGGACTACHVGGGTATCTAATCC) were used for PCR amplification of the V3-V4 hypervariable regions of the 16S rRNA genes (Klindworth et al., 2013). The amplified region was 390–410 bp long and an average of 67,000 reads per sample were obtained. The mixture of amplicons was sequenced using an Illumina MiSeq 2 × 250 v2 platform (Estonian Genome Centre, University of Tartu, Estonia).

### Taxonomic Profiling of Microbiota Samples

The DNA sequence data was analyzed using a BION-meta^1^, currently unpublished open source program, according to the author’s instructions. Sequences were first cleaned at both ends using a 99.5% minimum quality threshold for at least 18 of 20 bases for 5′-end and 28 of 30 bases for 3′-end, then joined, followed by the removal of contigs shorter than 350 bp. Then sequences were cleaned of chimeras and clustered by 95% oligonucleotide similarity (k-mer length of 8 bp, step size 2 bp). Lastly, consensus reads were aligned to the SILVA reference 16S rDNA database (v123) using a word length of 8 and similarity cut-off of 90%. The bacterial designation was analyzed at different taxonomic levels, down to species if applicable.

### Calculations

For quantitative data analysis, the relative data of bacterial abundances from 16S rDNA sequencing analysis were first converted to quantitative values [X_i_ (g/L), where i illustrates bacterial taxa i] by the formula: X_i_ = X_t_^∗^ A_i_, where X_t_ is the dry weight of the total biomass of bacteria (g/L) and A_i_ is the relative abundance of bacterial taxa i in the sample.

The growth characteristics of the bacteria in A-stat and De-stat experiments were calculated based on bacterial mass, total volume of medium pumped out from the fermenter (V_OUT_, L) and product concentrations in the culture medium (mol/L) as follows:

(1) $$\mu =\frac{d\,\left(V_{O\,U\,T}\right)}{V\times d\,t}+\frac{d\,\left(X_{t}\right)}{d\,t\times X_{t}}$$

(2) QS=iSi×d⁢(VO⁢U⁢T)V×Xt×d⁢t-d⁢(Si)d⁢t×Xt

(3) QP=iPi×d⁢(VO⁢U⁢T)V×Xt×d⁢t+d⁢(Pi)d⁢t×Xt

where μ is the specific growth rate (1/h), Q_S_ is the specific consumption rate of carbohydrate (in carbon equivalents, mol-C/g-X_t_/h), S_i_ is the concentration of consumed carbohydrate i (C-mol/L), Q_Pi_ is the specific production rate of product i (mol-prod/g-X/h), P_i_ is the concentration of product i (mol/L), V is the current fermenter volume (L), V_OUT_ is the outflow volume and t is the cultivation time (h).

### Statistical Analysis

Concentrations of metabolites or abundances of bacteria from three independent experiments were compared by average values and by unpaired and t-test (unadjusted *P*-values < 0.05 were considered significant) for chemostat point comparison. To compare differences in bacterial abundances and metabolite productions during A-stat and De-stat experiments, samples were divided into two groups: (1) samples taken at *D* < 0.07 1/h (slow growth) and (2) samples taken at *D* > 0.17 1/h (fast growth). Average and standard deviation of bacterial abundances and metabolite productions were calculated in both groups and a single parametric t-test was used to estimate statistical significance.

### Ethics Statement

This study was approved by the Tallinn Medical Research Ethics Committee, Estonia (protocol no. 554).

## Results

### Reproducibility of Chemostat Cultures of Fecal Microbiota

Until now, continuous fecal cultures have mostly been inoculated with fresh fecal inocula and information on reproducibility of this type of experiment is scarce. Hence, the aim of this work was to elucidate the reproducibility of the continuous fecal cultures. To elucidate the reproducibility of the growth of biological replicates of adult pooled fecal microbiota, six chemostat cultures (three at D_low_ = 0.05 1/h and three at D_high_ = 0.2 1/h) in a defined base medium with apple pectin and mucin were carried out.

#### Formation of Organic Acids and Gases

**FIGURE 2:**
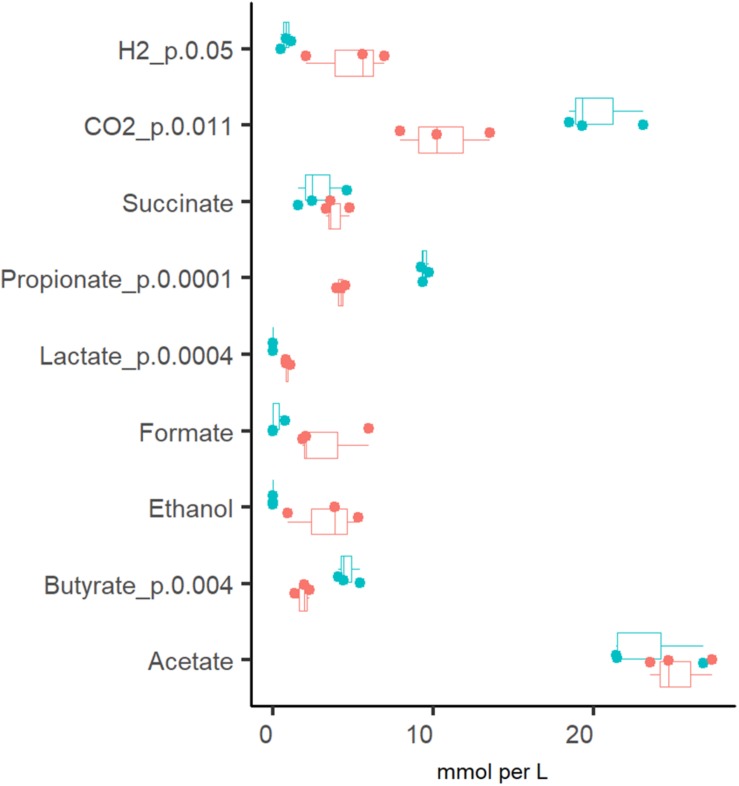
Comparison of the fermentation products from three (independent) chemostat experiments with the same pooled fecal culture at dilution rates 0.05 (blue dots) and 0.2 (red dots) 1/h in apple pectin containing medium. Numbers behind the metabolite name indicate significant difference between D_low_ and D_high_ (*D* = 0.05 and 0.2 1/h, respectively).

The production of organic acids and gases from pectin and mucin was related to the specific growth rate (Figure 2). The most abundant organic acid in all chemostat cultures was acetate [23.2 ± 3.1 and 25.2 ± 2.0 mM at D_low_ (0.05 1/h) and D_high_ (0.2 1/h), respectively]. Compared to D_high_, the production of propionate, butyrate and carbon dioxide was more enhanced at D_low_. At D_low_, 9.4 ± 0.3 mM propionate and less than 4 mM succinate were produced, while at D_high_, concentrations of propionate and succinate were practically equal (4.2 ± 0.3 and 3.9 ± 0.8 mM, respectively). The ratio of acetate, propionate and butyrate was 5:2:1 at D_low_ and 14:2:1 at D_high_. Similar to the product profile at D_low_, a ratio of 4:2:1 for acetate:propionate:butyrate was reported by Larsen et al. (2019) in TIM-2 experiments. In accordance with the organic acid profiles, about twice as much carbon dioxide was produced at D_low_ than at D_high_ (20.3 ± 2.4 and 10.5 ± 2.8 mmol per L medium, respectively). On average, the relative difference between parallels of the concentrations of propionate, butyrate and lactate in stabilized fecal cultures (after six residential times) remained below 10% and that of acetate below 25% at both dilution rates (D_low_ and D_high_).

**FIGURE 3:**
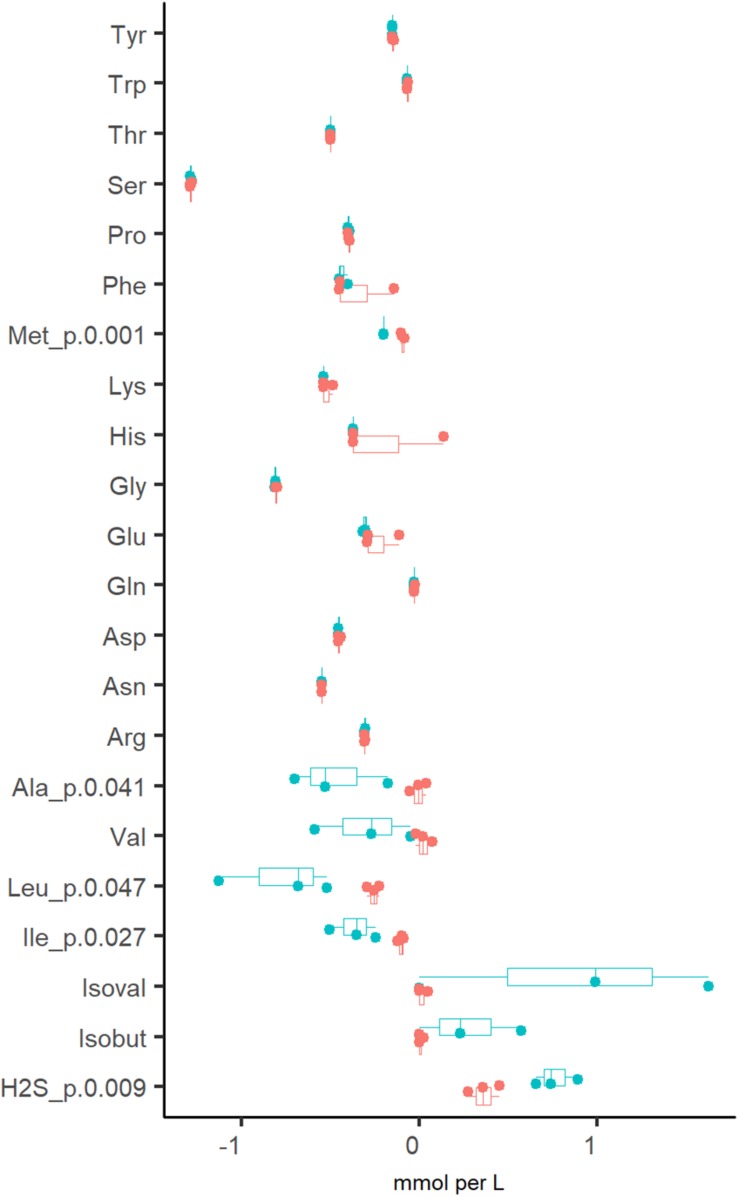
Comparison of amino acid consumptions and related metabolites (branched chain fatty acids and H_2_S) from three independent chemostat experiments of fecal culture at dilution rate 0.05 (blue dots) and 0.2 (red dots) 1/h in apple pectin medium. Positive values indicate the production and negative values indicate the consumption of the amino acid. Numbers behind the metabolite name indicate significant difference between D_low_ and D_high_ (*D* = 0.05 and 0.2 1/h, respectively). isoval, isovalerate; isobut, isobutyrate.

In addition to organic acids and gases, the consumption of amino acids was analyzed. It was determined that amino acids were fully depleted from the culture medium at both dilution rates, except for alanine and branched-chain amino acids (BCAA), which were practically not consumed at D_high_ (Figure 3). The increased production of propionate, butyrate and CO_2_ at D_low_ was accompanied by the conversion of BCAA to isobutyric (0.27 ± 0.29 mM) and isovaleric acids (0.87 ± 0.82 mM). Another amino acid degradation product significantly higher at D_low_ was H_2_S (0.76 ± 0.12 vs. 0.36 ± 0.09 mmol per L medium at D_low_ vs. D_high_, respectively) derived from the sulfur-containing amino acids Cys and Met. At D_high_, isoleucine and leucine were consumed in the range required for biomass formation (0.18–0.24 and 0.45–0.58 mmol/gDW, respectively. Supplementary Table S2 Chemostat), based on the amino acid contents in the biomass of *E. coli* (0.22 and 0.37 mmol/gDW for Ile and Leu, respectively; Valgepea et al., 2011) and *Lactococcus lactis* (0.25 and 0.37 mmol/gDW for Ile and Leu, respectively; Adamberg et al., 2012). As the total amount of biomass produced was 0.5–0.7 g/L (Supplementary Table S2 Chemostat), the consumption of other amino acids exceeded 1.6–5.5 times the amount required for biomass synthesis, except for serine, which was consumed about 10 times as much. Serine is the major amino acid in mucins and may be converted to acetate. However, serine degradation (2.1 and 2.6 mmol/gDW at D_low_ and D_high_, respectively) could not have contributed to more than 5% of the total acetate production (39 and 51 mmol/gDW at D_low_ vs. D_high_, respectively) as 80–86% of the carbon was derived from carbohydrate fermentation. The overall carbon recovery was 79% and 67% at D_low_ and D_high_, respectively, showing that some products were under-determined or missing, especially at high dilution rate.

#### Growth Rate Specific Differences of Fecal Microbiota

**FIGURE 4:**
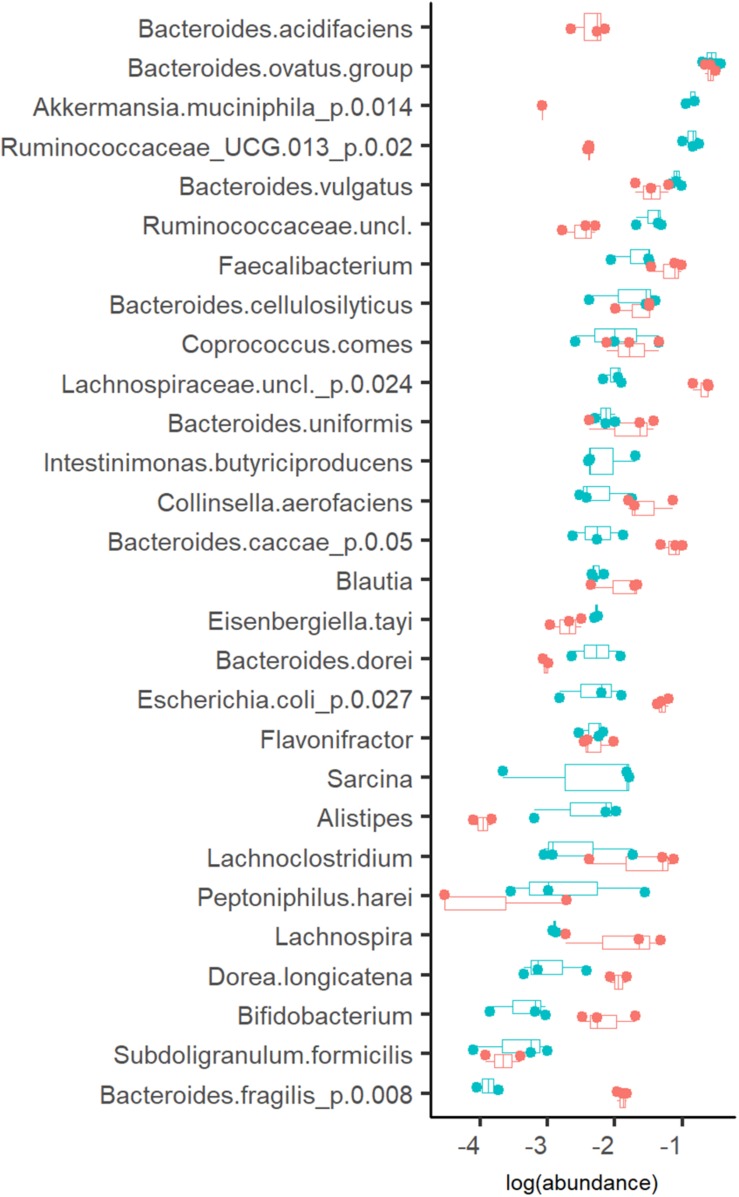
Abundance of bacteria (in log scale) at dilution rate 0.05 (blue dots) and 0.2 (red dots) 1/h in apple pectin medium. Each dot represents an independent chemostat experiment. Numbers behind the metabolite name indicate significant difference between D_low_ and D_high_ (*D* = 0.05 and 0.2 1/h, respectively).

The profiles of the metabolic products were in accordance with the bacterial compositions detected (Figures 2–4). Three taxa clearly prevalent at both dilution rates were the acetate- and propionate- or succinate-producing species *Bacteroides ovatus* (17 and 14%, at D_low_ and D_high_, respectively), *Bacteroides vulgatus* (7.9 and 3.6%, at D_low_ and D_high_, respectively) and butyrate-producing bacterium *Faecalibacterium* (2.4 and 7.2%, at D_low_ and D_high_, respectively). Also several other bacteria were abundant (1–4% of the total population) at both dilution rates, such as mixed acid (acetate, propionate and butyrate) fermenting *bacteria*, and the acetate- and propionate-producing *Bacteroides uniformis* and *Bacteroides cellulosilyticus* (Figure 4). At D_low_ the mucin degrading species *Akkermansia muciniphila* and a group of Ruminococcaceae UCG-013 (from 0.1 to 16% and from 0.5 to 14%, respectively) were significantly enriched. In different, at D_high_, *Bacteroides caccae*, Lachnospiraceae unclassified and mainly acetate-producing *E. coli* (7.7%, 21% and 6.3% of total reads, respectively) became dominant. The butyrate-producing bacterium *Intestimonas butyriciproducens* and *Sarcina* were detected only at D_low_, whereas *Bacteroides acidifaciens* was found only at D_high_. The increased production of ethanol and formate at D_high_ can be linked to higher abundances of *Dorea* and *Blautia*.

A remarkable enrichment of Enterobacteriaceae (up to 60% of total population) was observed in batch phase before starting the continuous flow. The amount of *E. coli* formed nearly 50% of the microbial population but decreased to about 10% in a chemostat stabilized at D_high_, and to 1.2% at D_low_. These data confirm the competitiveness of the fast-growing *E. coli* at high dilution rates.

### Comparison of A-Stat and De-Stat Cultures

Changes in the dilution rate in both directions between 0.05 to 0.2 1/h, starting from the stabilized cultures of pooled fecal microbiota in apple pectin and birch xylan media, were analyzed. In A-stat, the dilution rate was gradually increased from 0.05 to 0.2 1/h, and in De-stat the dilution rate was gradually decreased from 0.2 to 0.05 1/h. The stabilization of the chemostat culture was controlled by the titration rate of sodium hydroxide, indicating the rate of acid production rate, and the gas production rate (Supplementary Figure S1). Average fluctuations of these parameters below 5% within the last three residential times were considered to be stable cultures to start the changestat algorithm (on average, six or seven residential volumes were needed to achieve the stable cultures).

#### Formation of Organic Acids and Gases

**FIGURE 5:**
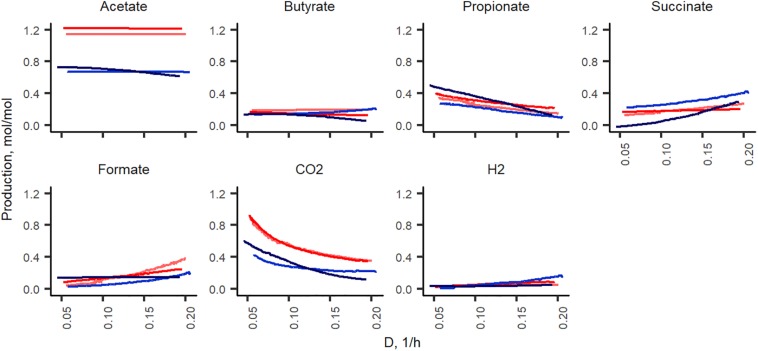
Fermentation product yields per carbohydrates consumed (mol/mol) during the growth in change-stat experiments. The fiber source is indicated by color (red, apple pectin; blue, birch xylan), and the color intensity refers to culture type: A-stat (light color) and De-stat (dark color). D – dilution rate (1/h).

In total, of one mole of carbohydrates, 1.2–1.3 and 1.7–1.9 mol of acids were produced in xylan and pectin supplemented media, respectively. Acetate formed in nearly two thirds of all fermentation products and its production did not depend on the dilution rate (Figure 5). Except for acetate and carbon dioxide, the formation of other metabolites from pectin and xylan was comparable in both directions of the dilution rate change (from 0.05 to 0.2 and from 0.2 to 0.05 1/h). Almost twice as much acetate was produced from xylan than from pectin (0.6–0.7 and 1.1–1.2 mol per mole of carbohydrates consumed, respectively). As xylose is a five-carbon sugar and galacturonic acid is a six-carbon compound, the lower amount of acids produced in the xylan medium can partly be explained by these differences. However, at all dilution rates, the carbon balance (C_substrates_ – C_products_) was still lower in the xylan- than in the pectin-containing medium (average values 74 ± 5% and 82 ± 2%, respectively). The formation of other metabolites, especially carbon dioxide and propionate, was strongly dilution rate-dependent. The synthesis of carbon dioxide was 0.9 and 0.4 mmol per L medium at D_low_ and at D_high_ in the pectin medium in both experimental directions (A-stat and De-stat). A similar trend was observed for xylan, suggesting that the production of carbon dioxide is linked to the growth rate rather than the substrate. To compensate for the change in the carbon flux caused by the decreased production of CO_2_, the succinate production increased, especially in the xylan medium. For example, in the A-stat experiment of the xylan medium, the reduction of CO_2_ production from 0.42 to 0.22 mol per mole of carbohydrates was compensated for by enhanced formation of succinate (0.26 to 0.42 at *D* = 0.05 1/h and *D* = 0.2 1/h, respectively) and formate (0.02 to 0.2 mol per mol carbohydrates at *D* = 0.05 1/h and *D* = 0.2 1/h, respectively). Similarly, propionate synthesis was decreased as a response to increasing formate production, keeping the carbon flux consistent (0.27 to 0.09 mol per mol carbohydrates at *D* = 0.05 1/h and *D* = 0.2 1/h, respectively). A reverse correlation between concentrations of propionic and succinic acids was observed. In comparing the gas production, notably less carbon dioxide (0.3–0.4 mol per mole of carbohydrates consumed) was formed from xylan, built of xylose, a five-carbon molecule, at all dilution rates. Carbon dioxide may originate from succinate to propionate conversion or butyrate production or demetoxylation of metoxylated galacturonic acid (Figure 5).

The described changes were characteristic under both directions of the dilution rate (A-stat and De-stat). These data suggest that the acceleration rate applied allowed the culture to adapt to the changing conditions.

#### Consumption and Formation of Amino Acids

Similarly to the chemostat cultures, most of the amino acids were completely depleted from the medium, except for alanine and BCAA (Table 1). The consumption of valine and leucine increased at lower dilution rates. The formation of isobutyrate and isovalerate was practically missing at high dilution rates, but they were produced in concentrations of 2.2–11 and 13–44 mmol/mol-carbohydrates, respectively, at low dilution rates (*D* < 1/h) (Table 1). In both media, the alanine metabolism was more intensive at low dilution rates (up to 14 mmol/mol-carbohydrates). The degradation of the reducing agent cysteine to H_2_S up to 41 mmol/mol-carbohydrates was observed at low dilution rates in all experiments (Table 1), which corresponds to the degradation of 22% of the total cysteine.

**TABLE 1:** Consumption of amino acids and formation of degradation products from amino acids (mmol per mol carbohydrates consumed) during A-stat and De-stat experiments and significantly different at fast or slow dilution rate.

Experiment*	State**	D, 1/h	Isobutyrate	Isovalerate	H_2_S	Ala	Ile	Leu
Xyl_A	SS	0.058	2.2	13.6	22.1	11.0	6.4	12.7
Xyl_D	Q	0.058	ND	ND	29.7	2.3	4.8	12.6
Xyl_D	SS	0.193	ND	23.9	7.4	ND	2.8	6.8
Xyl_A	Q	0.193	ND	1.0	12.4	3.4	4.1	5.5
Pec_A	SS	0.055	11.3	44.1	29.1	6.9	10.6	16.3
Pec_D	Q	0.054	2.7	17.1	41.3	14.3	8.8	15.2
Pec_D	SS	0.196	0.2	1.8	12.2	ND	5.7	8.5
Pec_A	Q	0.197	4.1	12.2	16.0	ND	5.4	2.2

*A and D indicate A-stat and De-stat, respectively. **SS and Q indicate steady state and quasi steady state, respectively. ND, not detected.

#### Growth Rate Specific Changes in Fecal Microbiota

**FIGURE 6:**
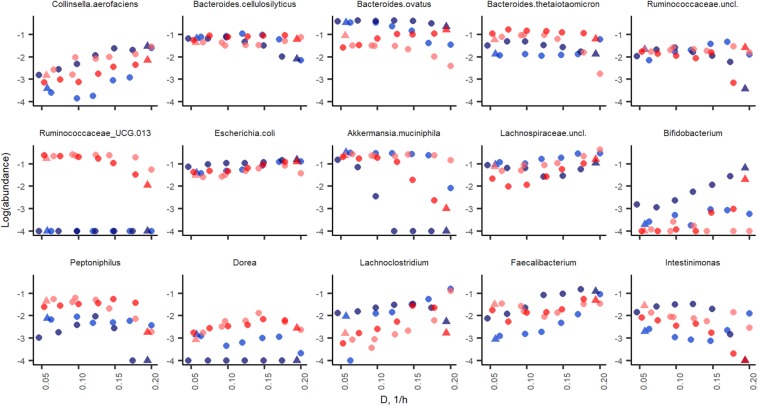
Bacterial taxa enriched in apple pectin (red) and xylan (blue) medium during A-stat (light color) and De-stat (dark color) experiments. Color indicates the fiber source in the medium (red, apple pectin; blue, birch xylan). D-dilution rate (1/h). Only taxa those of which abundance was changed during experiments are shown.

The initial fecal slurry contained 88 bacterial species with abundance above 0.1% and, of these, 25 species had abundance higher than 1% (Supplementary Table S1). During the chemostat and the following A-stat and De-stat cultivations, the species richness decreased to 27–32 and 10–18 species with abundance of 0.1 and 1% with apple pectin and xylan, respectively. A significant decrease in species richness has been shown by other authors (McDonald et al., 2013; Chung et al., 2016). The abundance of the majority of bacterial taxa was determined by the dilution rate on both substrates. The prevailing genus in the consortia – *Bacteroides* (up to 58% of the total population) – adapted well within the whole range of specific growth rates tested. However, the abundances of some species, such as *B. ovatus* and *Bacteroides cellulosilyticus*, tended to decrease at higher dilution rates (A-stat) in the xylan-supplemented medium (*p* = 0.02) (Figure 6). Pectin selectively enriched the Ruminococcaceae group UCG013, which was never detected in the xylan-containing medium. The abundance of the Ruminococcaceae group UCG-013 was also related to the dilution rate being 17% at lower dilution rates and down to 5% at dilution rates below *D* < 0.15 in both change directions (Figure 6). These data are in accordance with the chemostat results.

Significant increases in abundances of *Collinsella aerofaciens* (*p* = 0.002), *E. coli* (*p* = 0.001), *Faecalibacterium prausnitzii* (*p* = 0.009) and a group of Lachnospiraceae (closest similarity to *Coprococcus*, *p* = 0.008) (median abundances 1.6, 12, 5.1, and 19%, respectively) were observed at dilution rates above 0.17 1/h on both substrates. Although the abundances of butyrate-producing bacteria (*Faecalibacterium*, *Coprococcus*, and Lachnospiraceae) increased along with the increasing dilution rate, the tested substrates and conditions did not enhance the production of butyrate, resulting in other fermentation products instead.

At higher dilution rates, the increased formate production was accompanied by higher amounts of species from the genus *Lachnoclostridium* that are known to be involved in formate production. The abundance of the prevailing species at dilution rates below 0.07 1/h, *A. muciniphila* (median abundance 22%) decreased significantly at dilution rates above 0.17 1/h (abundance > 1%) in all changestat experiments. This is in accordance with the production of propionic acid, the characteristic metabolite of *Akkermansia* (Figure 5). Another taxa inhibited at higher dilution rates was *Intestinimonas* (Figure 6).

## Discussion

In continuous cultures, the environmental parameters, including substrate concentrations, pH, and flow rate, can be precisely controlled. The *in vivo* situation in the colon probably remains somewhere between chemostat and batch states, i.e., the availability of fermentable substrates decreases, the amounts of metabolites change dynamically and pH moves toward the alkaline region slowly. Our results demonstrate that the changestat techniques, the A-stat and De-stat, can be applied to study the effects of growth rate on the composition and metabolism of fecal microbiota. Using the same fecal inoculum, we showed that continuous cultures are reproducible at dilution rates of *D* = 0.2 and 0.05 1/h. The dilution rate during the stabilization phase impacts the results of the following culture characteristics. Therefore, the microbiota and metabolite patterns were comparatively analyzed in A-stat and De-stat cultures in a defined base medium containing mucin and either apple pectin or xylan. Similar microbiota and metabolite structures were observed within the scanned range of dilution rates in both directions, from *D* = 0.05 to 0.2 1/h or vice versa. The data of the steady state point of the chemostat at *D* = 0.2 1/h and the end point of the A-stat (*D* = 0.2 1/h) coincided well. Thus, the fecal culture was able to adapt to the change rate applied. This shows that by using suitable acceleration or deceleration rates it is possible to achieve a state of culture comparable to those of classical chemostat cultures. This is new information for consortia cultivation, although it has long been known for pure cultures.

In a previous De-stat study with fecal samples from children (5–15 years old), similar structural changes in the fecal microbiota were seen (Adamberg and Adamberg, 2018). This suggests that for this age group adult-like microbiota are mostly established. Moreover, these data indicate the crucial role of the growth rate in metabolism and the structure of colon microbiota. For example, in both studies, the taxa clearly preferring high dilution rates were *C. aerofaciens*, *Bifidobacterium*, *B. vulgatus*, *E. coli*, *Lachnospira* and *Lachnoclostridium*, whereas *A. muciniphila* and the Ruminococcaceae group UCG-013 were enriched at low dilution rates. Accordingly, *in vivo* studies have shown that *Akkermansia* and ruminococci are more prevalent in people with slow colonic transit, while *Bifidobacterium* and *Lachnospiraceae* correlate with high transit rates (Kim et al., 2015; Roager et al., 2016; Vandeputte et al., 2016). The impact of pectin structure on the dynamics metabolism and fecal microbiota has been shown by Larsen et al. (2019). Highly methoxylated pectins were shown to stimulate *F. prausnitzii*, commonly referred to as a health-promoting species. In both of our studies with apple pectin, the abundance of *Faecalibacterium* was 1-7% of the total population within the whole range of dilution rates, indicating the importance of pectin for the growth of colonic *Faecalibacterium* (the current study, and Adamberg and Adamberg, 2018).

Similarities between child and adult fecal pools were also observed at the metabolic level, but with some minor differences. Although the abundances of *Faecalibacterium* in adult and child cultures were similar (1.9–5.2% and 4.5–7.5%, respectively), about twice as much butyrate and CO_2_ were produced by adults’ than by children’s consortia at slow dilution rates, suggesting higher activity of the butyrate producers in adult microbiota. The dynamics of other metabolites, including BCFA and H_2_S, from the degradation of amino acids was comparable in both fecal consortia. The enhanced production of propionate, as well as the extensive use of amino acids and BCFA formation at low specific growth rates, may be related to a shortage of energy, ammonia, or NAD^+^ regeneration. These properties are known for pure cultures (Tempest, 1984) but seem to also be common for fecal microbial consortia (Adamberg and Adamberg, 2018). K_s_ values of carbohydrates and amino acids for different species should be measured to determine the carbon or nitrogen limitation, but these data are very scarce for gut bacteria.

The application of changestat makes it possible to elucidate the mechanisms of the co-existence of different bacteria by adapting mixed cultures under different environmental conditions. The cultivation of microbial consortia instead of single cultures is a promising approach for several biotechnological applications, including the development of multi-strain probiotics or material for fecal transplantation. As mentioned above, this and previous studies reveal that various consortia can be generated using continuous cultivation strategies. Still, the question remains of how to produce safe consortia with desired properties and/or therapeutic effects. Thus, detailed information is needed about selective pressure on the development of bacterial consortia under various environmental conditions: pH, the availability and concentration of substrate, dilution rate, selective additives, defining the inocula, etc. The changestat approach makes it possible to scan selected environmental conditions in an adaptive manner; providing an opportunity to predict appropriate conditions for the development of a consortium with a desired bacterial pattern.

## Conclusion

The changestat experiments presented in this paper showed that continuous cultures of complex fecal consortia are reproducible in chemostat. Similar microbiota and metabolite changes were observed within the scanned range of dilution rates in changestat cultures in both directions, from *D* = 0.05 to 0.2 1/h or vice versa. This is new information for consortia cultivation, although it has long been known for pure cultures.

Our work confirmed that dilution rate is a crucial trigger in consortia development. Some species, such as propionate-producing *B. ovatus* and *B. vulgatus* and butyrate-producing *Faecalibacterium*, were prevalent within the whole range of dilution rates, while the mucin-degrading bacterium *A. muciniphila* and some ruminococci were enriched at low dilution rates only.

The production of organic acids and gases from pectin in the presence of mucin was related to the dilution rate in chemostat cultures. The ratio of acetate, propionate and butyrate was 5:2:1 at *D* = 0.5 1/h and 14:2:1 at *D* = 0.2 1/h. Most amino acids were completely depleted from the medium except for alanine and BCAA, which were metabolized to isobutyric and isovaleric acids in chemostat as well as changestat cultures.

For further analysis of the interactions in complex consortia, other gut-relevant environmental conditions and substrates available in the colon will be studied. It should be stressed that, in addition to high-throughput sequencing analysis, it is necessary to concentrate on the growth and metabolism of fecal consortia to work out novel methods for bacterial therapies. Changestat cultures make it possible to screen the combined effects of important environmental and feed parameters, such as acidity, temperature, medium composition, dilution rate, and the effects of inocula and multiple substrates.

## Data Availability Statement

The datasets analyzed for this study can be found in the European Nucleotide Archive on the site PRJEB33931 (ERP116764, dataset name “Run26_cult2”).

## Ethics Statement

The studies involving human participants were reviewed and approved by the Tallinn Medical Research Ethics Committee, Estonia (protocol No. 554). The patients/participants provided their written informed consent to participate in this study.

## Author Contributions

KA and SA designed the study. KA, GR, and SA carried out the experiments and data analyses. KA drafted the first manuscript. GR and SA contributed by writing and editing the final manuscript. All authors approved the submitted version.

## Conflict of Interest

The authors declare that the research was conducted in the absence of any commercial or financial relationships that could be construed as a potential conflict of interest.

## Footnotes

1 www.box.com/bion

## Supplementary Material

FIGURE S1 On-line measured data that are used for stability analyses during chemostat before A-stat (left side figures) or De-stat (right side figures) experiments. Upper figures show data in xylan + mucin medium and lower figures in apple pectin medium. Base rate, titration rate of 1M NaOH (ml/min); rGas, gas production rate (ml/min); D, dilution rate (1/h).

TABLE S1 Abundances of bacteria in the inocula and samples from A-stat and De-stat experiments.

TABLE S2 Production of metabolites per carbohydrate consumed (mol/mol-carbohydrates and mmol/gDW), carbon recovery, and biomass yield (gDW/g-carbohydrates) during A-stat and De-stat experiments.

## References

[B1] Adamberg K. Adamberg S. (2018). Selection of fast and slow growing bacteria from fecal microbiota using continuous culture with changing dilution rate. *Microb. Ecol. Health Dis.* 29:1549922. 10.1080/16512235.2018.1549922 30532686 PMC6282430

[B2] Adamberg K. Kolk K. Jaagura M. Vilu R. Adamberg S. (2018). The composition and metabolism of faecal microbiota is specifically modulated by different dietary polysaccharides and mucin: an isothermal microcalorimetry study. *Benef. Microbes* 9 21–34. 10.3920/BM2016.0198 29022389

[B3] Adamberg K. Seiman A. Vilu R. (2012). Increased biomass yield of *Lactococcus lactis* by reduced overconsumption of amino acids and increased catalytic activities of enzymes. *PLoS One.* 7:e48223. 10.1371/journal.pone.0048223 23133574 PMC3485057

[B4] Adamberg K. Valgepea K. Vilu R. (2015). Advanced continuous cultivation methods for systems microbiology. *Microbiolgy* 161 1707–1719. 10.1099/mic.0.000146 26220303

[B5] Aguirre M. Eck A. Koenen M. E. Savelkoul P. H. M. Budding A. E. Venema K. (2015). Evaluation of an optimal preparation of human standardized fecal inocula for in vitro fermentation studies. *J. Microbiol. Methods* 117 78–84. 10.1016/j.mimet.2015.07.019 26222994

[B6] Brahma S. Martínez I. Walter J. Clarke J. Gonzalez T. Menon R. (2017). Impact of dietary pattern of the fecal donor on in vitro fermentation properties of whole grains and brans. *J. Funct. Foods* 29 281–289. 10.1016/j.jff.2016.12.042

[B7] Burkitt D. P. Walker A. R. P. Painter N. S. (1972). Effect of dietary fibre on stools and transit-times, and its role in the causation of disease. *Lancet* 30 1408–1411. 10.1016/s0140-6736(72)92974-1 4118696

[B8] Bussolo de Souza C. Jonathan M. Saad S. M. I. Schols H. A. Venema K. (2019). Degradation of fibres from fruit by-products allows selective modulation of the gut bacteria in an in vitro model of the proximal colon. *J. Funct. Foods* 57 275–285. 10.1016/j.jff.2019.04.026

[B9] Chung W. S. F. Walker A. W. Louis P. Parkhill J. Vermeiren J. Bosscher D. (2016). Modulation of the human gut microbiota by dietary fibres occurs at the species level. *BMC Biol.* 14:3. 10.1186/s12915-015-0224-3 26754945 PMC4709873

[B10] Chung W. S. F. Walker A. W. Vermeiren J. Sheridan P. O. Bosscher D. Garcia-Campayo V. (2019). Impact of carbohydrate substrate complexity on the diversity of the human colonic microbiota. *FEMS Microbiol. Ecol.* 95:fiy201. 10.1093/femsec/fiy201 30304332 PMC6238074

[B11] Cummings J. H. Jenkins D. J. A. Wiggins H. S. (1976). Measurement of the mean transit time of dietary residue through human gut. *Gut* 17 210–218. 10.1136/gut.17.3.210 1269989 PMC1411154

[B12] Fallingborg J. Christensen L. A. Ingeman-Nielsen M. Jacobsen B. A. Abildgaard K. Rasmussen H. H. (1989). PH-profile and regional transit times of the normal gut measured by a radiotelemetry device. *Aliment. Pharmacol. Ther.* 3 605–613. 2518873 10.1111/j.1365-2036.1989.tb00254.x

[B13] Freter R. Stauffer E. Cleven D. Holdeman L. Moore W. E. C. (1983). Continuous-flow cultures as in vitro models of the ecology of large intestinal flora. *Infect. Immun.* 39 666–675. 10.1128/iai.39.2.666-675.1983 6339387 PMC348003

[B14] Gottschal J. C. (1990). Different types of continuous culture in -ecological studies. *Methods Microbiol.* 22 87–124. 10.1016/s0580-9517(08)70240-x 7092201

[B15] Jorup-Rönström C. Håkanson A. Sandell S. Edvinsson O. Midtvedt T. Persson A. K. (2012). Fecal transplant against relapsing Clostridium difficile-associated diarrhea in 32 patients. *Scand. J. Gastroenterol.* 47 548–552. 10.3109/00365521.2012.672587 22468996

[B16] Kasemets K. Drews M. Nisamedtinov I. Adamberg K. Paalme T. (2003). Modification of a-stat for the characterization of microorganisms. *J. Microbiol. Methods* 55 187–200. 10.1016/S0167-7012(03)00143-X 14500010

[B17] Kim S. E. Choi S. C. Park K. S. Park M. I. Shin J. E. Lee T. H. (2015). Change of fecal flora and effectiveness of the short-term VSL#3 probiotic treatment in patients with functional constipation. *J. Neurogastroenterol. Motil.* 21 111–120. 10.5056/jnm14048 25537674 PMC4288088

[B18] Klindworth A. Pruesse E. Schweer T. Peplies J. Quast C. Horn M. (2013). Evaluation of general 16S ribosomal RNA gene PCR primers for classical and next-generation sequencing-based diversity studies. *Nucleic Acids Res.* 41 1–11. 10.1093/nar/gks808 22933715 PMC3592464

[B19] Lagier J.-C. Armougom F. Million M. Hugon P. Pagnier I. Robert C. (2012). Microbial culturomics: paradigm shift in the human gut microbiome study. *Clin. Microbiol. Infect.* 18 1185–1193. 10.1111/1469-0691.12023 23033984

[B20] Langendijk P. Schut F. Jansen G. Raangs G. Kamphuis G. Wilkinson M. (1995). Quantitative fluorescence in situ hybridization of *Bifidobacterium* spp. with genus-specific 16S rRNA-targeted probes and its application in fecal samples. *Appl. Environ. Microbiol.* 61 3069–3075. 7487040 10.1128/aem.61.8.3069-3075.1995 PMC167584

[B21] Larsen N. Bussolo de Souza C. Krych L. Cahú T. B. Wiese M. Kot W. (2019). Potential of pectins to beneficially modulate the gut microbiota depends on their structural properties. *Front. Microbiol.* 10:223. 10.3389/fmicb.2019.00223 30828323 PMC6384267

[B22] Macfarlane S. Quigley M. E. Hopkins M. J. Newton D. F. Macfarlane G. T. (1998). Polysaccharide degradation by human intestinal bacteria during growth under multi-substrate limiting conditions in a three-stage continuous culture system. *FEMS Microbiol. Ecol.* 26 231–243. 10.1016/S0168-6496(98)00039-7

[B23] McDonald J. A. K. Schroeter K. Fuentes S. Heikamp-deJong I. Khursigara C. M. de Vos W. M. (2013). Evaluation of microbial community reproducibility, stability and composition in a human distal gut chemostat model. *J. Microbiol. Methods* 95 167–174. 10.1016/j.mimet.2013.08.008 23994646

[B24] Miller T. L. Wolin M. J. (1981). Fermentation by the human large intestine microbial community in an in vitro semicontinuous culture system. *Appl. Environ. Microbiol.* 42 400–407. 10.1128/aem.42.3.400-407.1981 7027952 PMC244027

[B25] Paalme T. Kahru A. Elken R. Vanatalu K. Tiisma K. Vilu R. (1995). The computer-controlled continuous culture of *Escherichia Coli* with smooth change of dilution rate (A-Stat). *J. Microbiol. Methods* 24 145–153. 10.1016/0167-7012(95)00064-x

[B26] Petrof E. O. Gloor G. B. Vanner S. J. Weese S. J. Carter D. Daigneault M. C. (2013). Stool substitute transplant therapy for the eradication of clostridium difficile infection: ‘RePOOPulating’ the gut. *Microbiome* 1:3. 10.1186/2049-2618-1-3 24467987 PMC3869191

[B27] Rajilić-Stojanović M. Heilig H. G. H. J. Molenaar D. Kajander K. Surakka A. Smidt H. (2009). Development and application of the human intestinal tract chip, a phylogenetic microarray: analysis of universally conserved phylotypes in the abundant microbiota of young and elderly adults. *Environ. Microbiol.* 11 1736–1751. 10.1111/j.1462-2920.2009.01900.x 19508560 PMC2784037

[B28] Roager H. M. Hansen L. B. S. Bahl M. I. Frandsen H. L. Carvalho V. Gøbel R. J. (2016). Colonic transit time is related to bacterial metabolism and mucosal turnover in the gut. *Nat. Microbiol.* 1:16093. 10.1038/nmicrobiol.2016.93 27562254

[B29] Rowland I. Gibson G. Heinken A. Scott K. Swann J. Thiele I. (2018). Gut microbiota functions: metabolism of nutrients and other food components. *Eur. J. Nutr.* 57 1–24. 10.1007/s00394-017-1445-8 28393285 PMC5847071

[B30] Russell J. B. Baldwin R. L. (1979). Comparison of substrate affinities among several rumen bacteria: a possible determinant of rumen bacterial competition. *Appl. Environ. Microbiol.* 37 531–536. 10.1128/aem.37.3.531-536.1979 16345358 PMC243250

[B31] Sender R. Fuchs S. Milo R. (2016). Are we really vastly outnumbered? Revisiting the ratio of bacterial to host cells in humans. *Cell* 164 337–340. 10.1016/j.cell.2016.01.013 26824647

[B32] Sghir A. Chow J. M. Mackie R. I. (1998). Continuous culture selection of bifidobacteria and lactobacilli from human faecal samples using fructooligosaccharide as selective substrate. *J. Appl. Microbiol.* 85 769–777. 10.1111/j.1365-2672.1998.00590.x 9812388

[B33] Tannock G. W. (2017). *Understanding Bowel Bacteria.* Hoboken, NJ: John Wiley & Sons, Inc.

[B34] Tempest D. (1984). The status of YATP and maintenance energy as biologically interpretable phenomena. *Annu. Rev. Microbiol.* 38 459–486. 10.1146/annurev.micro.38.1.459 6388498

[B35] Valgepea K. Adamberg K. Vilu R. (2011). Decrease of energy spilling in *Escherichia coli* continuous cultures with rising specific growth rate and carbon wasting. *BMC Syst. Biol.* 5 106. 10.1186/1752-0509-5-106 PMC3149000 21726468

[B36] Vandeputte D. Falony G. Vieira-Silva S. Tito R. Y. Joossens M. Raes J. (2016). Stool consistency is strongly associated with gut microbiota richness and composition. Enterotypes and bacterial growth rates. *Gut* 65 57–62. 10.1136/gutjnl-2015-309618 PMC4717365 26069274

[B37] von Martels J. Z. H. Sadabad M. S. Bourgonje A. R. Blokzijl T. Dijkstra G. Faber K. N. (2017). The role of gut microbiota in health and disease: *in vitro* modeling of host-microbe interactions at the aerobe-anaerobe interphase of the human gut. *Anaerobe* 44 3–12. 10.1016/j.anaerobe.2017.01.001 28062270

[B38] Yen S. McDonald J. A. K. Schroeter K. Oliphant K. Sokolenko S. Blondeel E. J. M. (2015). Metabolomic analysis of human fecal microbiota: a comparison of feces-derived communities and defined mixed communities. *J. Proteome Res.* 14 1472–1482. 10.1021/pr5011247 25670064

