# Supplementary figures and images for: Use of Changestat for Growth Rate Studies of Gut Microbiota
Source: Front Bioeng Biotechnol. 2020 Feb 7;8:24. doi: 10.3389/fbioe.2020.00024 (PMC7019180; doi:10.3389/fbioe.2020.00024)

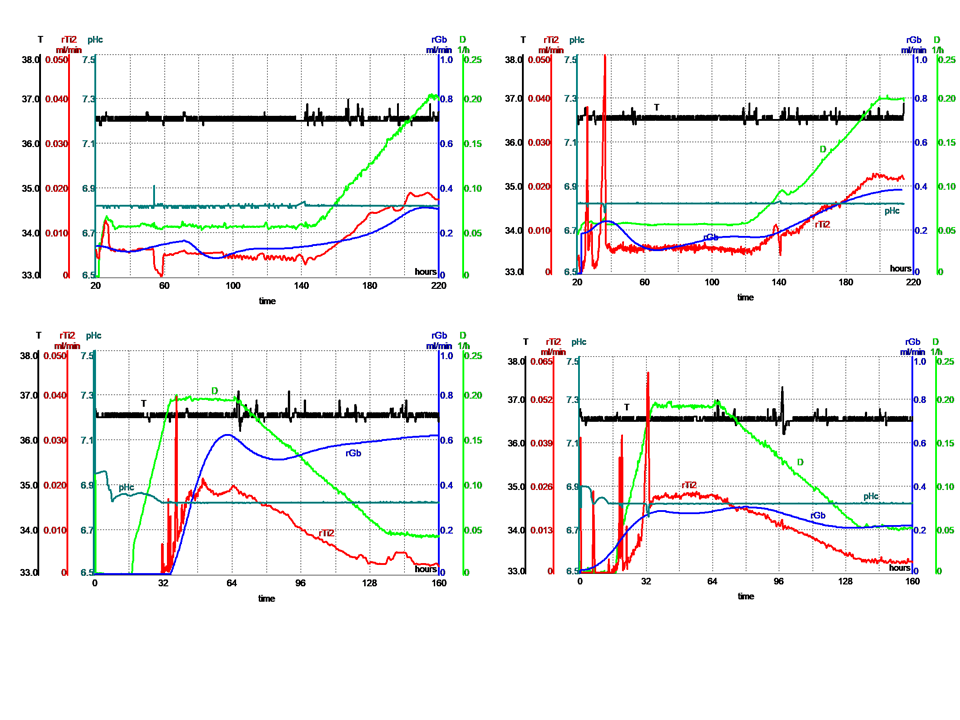

Supplement: FIGURE S1 — On-line measured data that are used for stability analyses during chemostat before A-stat (left side figures) or De-stat (right side figures) experiments. Upper figures show data in xylan + mucin medium and lower figures in apple pectin medium. Base rate, titration rate of 1M NaOH (ml/min); rGas, gas production rate (ml/min); D, dilution rate (1/h). [file Image_1.tif]
